# Supplementary material for: Phylogeny of Crepidotus applanatus Look-Alikes Reveals a Convergent Morphology Evolution and a New Species C. pini
Source: J Fungi (Basel). 2022 May 7;8(5):489. doi: 10.3390/jof8050489 (PMC9144983; doi:10.3390/jof8050489)
Supplement: Supplementary file 1 [file jof-08-00489-s001.zip › Table S1.pdf]

Table S1: Details on the studied specimens of *Crepidotus* (arranged within Slovakia from west to east). Specimens with asterisk symbol were sequenced.

Taxon	Collection number	Country, region/area	Locality	Habitat	Substrate	Coordinates	Elevation (m a.s.l.)	Date, collector
Crepidotus applanatus	SLO 1492*	**Slovakia,** Kremnické vrchy Mts.	Badín village, ca. 5 km NW of the church, the National Nature Reserve Badínsky prales	old growth forest of *Fagus sylvatica*, *Abies alba*	on wood of fallen trunk of *Fagus sylvatica*, 4th decay stage	48°41'12''N, 19°03'07''E	750	9.IX.2015, S. Jančovičová
	SLO 2551*	**Slovakia,** Veľká Fatra Mts.	Dolný Harmanec village, ca. 3.5 km NNW of the church, Rabkina skala rock	forest of *Fagus sylvatica* and mixed beech forest	on wood of fallen trunk of deciduous tree, 4th decay stage	48°50'29''N, 19°02'30''E	900	28.IX.2004, S. Adamčík
	SLO 2517	**Slovakia,** Vihorlatské vrchy Mts.	Jovsa village, ca. 1.5 km NE of the church, the National Nature Reserve Jovsianska hrabina	forest of *Carpinus betulus*, *Quercus robur*, *Q. petraea*	on wood of fallen trunk of *Quercus*, 4th decay stage	48°49'22''N, 22°06'27''E	170	18.IX.2001, S. Jančovičová
	SLO 1821*	**Slovakia,** Vihorlatské vrchy Mts.	Valaškovce military district, ca. 4 km SE of the former village of Valaškovce, the National Nature Reserve Vihorlat	forest of *Fagus sylvatica* and mixed beech forest	on wood of fallen trunk of *Fagus sylvatica*, 4th decay stage	48°53'22''N, 22°06'51''E	850	27.IX.2011, S. Jančovičová
	SLO 2524	**Slovakia,** Vihorlatské vrchy Mts.	Zemplínske Hámre village, ca. 4 km S of the church, N edge of the peatbog Hypkania	forest of *Fagus sylvatica* and mixed beech forest	on wood of fallen trunk of *Fagus sylvatica*, 4th decay stage	48°54'51''N, 22°09'29''E	830	17.IX.2003, S. Jančovičová
	SLO 2523	**Slovakia,** Vihorlatské vrchy Mts.	Zemplínske Hámre village, ca. 3.5 km SEE of the church	forest of *Fagus sylvatica* and mixed beech forest	on wood of fallen trunk of *Acer pseudoplatanus*, 3rd decay stage	48°55'11''N, 22°09'44''E	800	20.IX.2001, J. Vatava
	SLO 2534*	**Slovakia,** Vihorlatské vrchy Mts.	Remetské Hámre village, ca. 8 km N of the church, the Nature Reserve Jedlinka	forest of *Fagus sylvatica*, *Abies alba*	on wood of fallen trunk of *Fagus sylvatica*, 4th decay stage	48°54'51''N, 22°10'49''E	800	19.IX.2003, S. Jančovičová
	SLO 2535	**Slovakia,** Vihorlatské vrchy Mts.	Remetské Hámre village, ca. 8 km N of the church, the Nature Reserve Jedlinka	forest of *Fagus sylvatica*, *Abies alba*	on wood of fallen trunk of *Fagus sylvatica*, 4th decay stage	48°54'51''N, 22°10'49''E	800	19.IX.2003, S. Jančovičová
	SLO 2537*	**Slovakia,** Vihorlatské vrchy Mts.	Remetské Hámre village, ca. 8 km N of the church, the Nature Reserve Jedlinka	forest of *Fagus sylvatica*, *Abies alba*	on wood of fallen trunk of *Fagus sylvatica*, 4th decay stage	48°54'51''N, 22°10'49''E	800	19.IX.2003, S. Jančovičová
	SLO 2538	**Slovakia,** Vihorlatské vrchy Mts.	Remetské Hámre village, ca. 8 km N of the church, the Nature Reserve Jedlinka	forest of *Fagus sylvatica*, *Abies alba*	on wood of fallen trunk of *Fagus sylvatica*, 4th decay stage	48°54'51''N, 22°10'49''E	800	19.IX.2003, S. Jančovičová
	SLO 2539*	**Slovakia,** Vihorlatské vrchy Mts.	Remetské Hámre village, ca. 8 km N of the church, the Nature Reserve Jedlinka	forest of *Fagus sylvatica*, *Abies alba*	on wood of fallen trunk of *Fagus sylvatica*, 4th decay stage	48°54'51''N, 22°10'49''E	800	19.IX.2003, S. Jančovičová
	SLO 2533*	**Slovakia,** Vihorlatské vrchy Mts.	Remetské Hámre village, ca. 7.5 km N of the church, the Nature Monument Malé Morské oko	forest of *Fagus sylvatica*, *Abies alba*	on wood of fallen trunk of *Fagus sylvatica*, 4th decay stage	48°54'45''N, 22°10'59''E	750	19.IX.2003, S. Jančovičová
	SLO 2545	**Slovakia,**	Remetské Hámre village, ca. 1 km	acidophilous *Fagus sylvatica*	on wood of fallen trunk of	48°50'54''N	300–350	19.IX.2001,

		Vihorlatské vrchy Mts.	E of the church, the Nature Reserve Machnatý vrch	forest	*Fagus sylvatica*, 4th decay stage	22°11'24"E		S. Jančovičová
	SLO 2525	**Slovakia,** Vihorlatské vrchy Mts.	Remetské Hámre village, ca. 8 km NNE of the church, the National Nature Reserve Morské oko	forest of *Fagus sylvatica*, *Abies alba*	on wood of standing trunk of *Fagus sylvatica*, 4th decay stage	48°54'54"N, 22°12'06"E	650	12.VII.2001, S. Jančovičová
	SLO 2528*	**Slovakia,** Vihorlatské vrchy Mts.	Remetské Hámre village, ca. 8 km NNE of the church, the National Nature Reserve Morské oko	forest of *Fagus sylvatica*, *Abies alba*	on wood of fallen trunk of *Fagus sylvatica*, 4th decay stage	48°54'54"N, 22°12'06"E	650	12.VII.2001, S. Jančovičová
	SLO 2531	**Slovakia,** Vihorlatské vrchy Mts.	Remetské Hámre village, ca. 8 km NNE of the church, the National Nature Reserve Morské oko	forest of *Fagus sylvatica*, *Abies alba*	on wood of fallen branch of *Fagus sylvatica*, 3rd decay stage	48°54'54"N, 22°12'06"E	650	12.VII.2001, S. Jančovičová
	SLO 2532	**Slovakia,** Vihorlatské vrchy Mts.	Remetské Hámre village, ca. 8 km NNE of the church, the National Nature Reserve Morské oko	forest of *Fagus sylvatica*, *Abies alba*	on wood of fallen trunk of *Fagus sylvatica*, 4th decay stage	48°54'54"N, 22°12'06"E	650	12.VII.2001, S. Jančovičová
	SLO 2547*	**Slovakia,** Vihorlatské vrchy Mts.	Strihovce village, ca. 1.5 km SW of the church	forest of *Betula pendula*, *Populus tremula*, *Salix caprea*	on wood of fallen trunk of *Salix*, 4th decay stage	48°53'52"N, 22°15'31"E	550	18.IX.2003, S. Jančovičová
	SLO 1926	**Slovakia,** Bukovské vrchy Mts.	Nová Sedlica village, ca. 3 km NNE of the church, the National Nature Reserve Stužica	old growth forest of *Fagus sylvatica*, *Abies alba*	on wood of fallen trunk of *Fagus sylvatica*, 4th decay stage	49°04'42"N, 22°32'07"E	750	6.X.2016, S. Jančovičová
	SLO 2581*	**Norway,** Trøndelag county	Flatanger village, Stordalen	boreal rain forest of *Picea abies*	on wood of fallen trunk of cf. *Picea abies*, 4th decay stage	64°26'39"N, 10°58'12"E	60	4.IX.2009, V. Kučera
Crepidotus crocophyllus	SLO 2588*	**Slovakia,** Záhorská nížina Lowland	Malé Leváre village, ca. 3 km SW of the church, in the alluvium of the Morava River	mixed oak-elm-ash alluvial forest; *Quercus robur*, *Ulmus*, *Fraxinus*	on wood of fallen trunk of deciduous tree, 4th decay stage	48°31'12"N 16°56'39"E	150	7.V.2004, S. Jančovičová
	SLO 2433*	**Slovakia,** Záhorská nížina Lowland	Záhorie military district, Hlboké village, ca. 100 m NE of the hunters' cottage Šranek	forest of *Quercus robur*, *Pinus sylvestris*, *Betula pendula*	on wood of fallen trunk of deciduous tree, 4th decay stage	48°38'23"N, 17°21'15"E	200	27.V.2019, S. Jančovičová
	SLO 2590*	**Slovakia,** Devínska Kobyla Hills	Bratislava city, the borough of Devín, the Fialková dolina Nature Reserve	forest of *Acer campestre*, *A. platanoides*, *A. pseudoplatanus*, *Tilia cordata*, *T. platyphyllos*	on wood of fallen trunk of *Tilia*, 4th decay stage	48°10'10.8"N, 17°00'19.6"E	195	15.VI.2007, S. Jančovičová
	SLO 2553*	**USA, North Carolina,** Appalachian Mts.	SSW of the Asheville city, the Pisgah National Forest, the Bent Creek Experimental Forest	upland hardwood forest	on wood of fallen branch of deciduous tree, 4th decay stage	35°28'36"N, 82°39'47"W	900	16.VII.2004, S. Adamčík
Crepidotus ehrendorferi	SLO 2558*	**Slovakia,** Biele Karpaty Mts.	Nová Bošáca village, ca. 1.5 km NNE of the church, the Nature Monument Grúň	forest of *Quercus*, *Carpinus betulus*, *Fagus sylvatica*	on wood of stump of *Carpinus betulus*, 3rd decay stage	48°53'39"N, 17°47'57"E	440	18.X.2001, S. Jančovičová
	SLO 2587*	**Slovakia,** Považský Inovec Mts.	Moravany nad Váhom village, ca. 250 m SEE of the recreation centre Výtoky	forest of *Fraxinus excelsior*, *Acer*, *Carpinus betulus*	on wood of fallen branch of deciduous tree, 3rd decay stage	48°36'09"N, 17°56'14"E	300	29.IX.2012, J. Červenka
	SLO 707*	**Slovakia,** Zvolenská kotlina Basin	Zvolen city, ca. 3 km N of the city centre, Borová hora Arboretum	forest of *Tilia cordata*, *Quercus robur*, *Acer campestre*	on wood of fallen branch of *Tilia cordata*, 3rd decay stage	48°35'56"N, 19°08'01"E	300	28.IX.2009, S. Glejdura

Crepidotus malachioides	SLO 2578*	**Slovakia,** Podunajská nížina Lowland	Bratislava city, the borough of Petržalka, ca. 1 km SE of the Lafranconi bridge	mixed ash-alder alluvial forest; *Poulus nigra*, *Salix alba*, *Fraxinus*	on wood of fallen trunk of *Fraxinus excelsior*, 4th decay stage	48°07'49.5"N, 17°04'58.7"E	140	21.IX.2020, S. Jančovičová
	SLO 2391*	**Slovakia,** Východoslovenská nížina Lowland	Malé Raškovce village, ca. 2 km SE of the church, the Nature Reserve Raškovský luh	mixed oak-elm-ash alluvial forest; *Quercus robur*, *Acer campestre*, *Populus nigra*	on wood of fallen trunk of *Ulmus*, 4th decay stage	48°34'0.65"N, 21°56'28.04"E	100	28.V.2018, M. Lazor
	SLO 2561*	**Slovakia,** Slanské vrchy Mts.	Slanská Huta village, ca. 2 km SW of the church, the Nature Reserve Marocká hoľa	old growth forest of *Fagus sylvatica*	on wood of fallen trunk of *Fagus sylvatica*, 4th decay stage	48°35'23"N, 21°26'34"E	600	26.V.2019, M. Lazor
	SLO 1932*	**Slovakia,** Bukovské vrchy Mts.	Nová Sedlica village, ca. 3 km NNE of the church, the National Nature Reserve Stužica	old growth forest of *Fagus sylvatica*, *Abies alba*	on wood of fallen trunk of *Fagus sylvatica*, 4th decay stage	49°04'42"N, 22°32'07"E	750	6.X.2016, I. Tomášeková
Crepidotus malachius	SLO 2552*	**Slovakia,** Malé Karpaty Mts.	Sološnica village, ca. 3.5 km SE of the church, Sološnická dolina valley	forest of *Fagus sylvatica* and mixed beech forest	on wood of fallen trunk of *Acer campestre*, 3rd decay stage	48°26'47"N, 17°15'33"E	300	15.VII.2010, V. Kučera
	SLO 1497*	**Slovakia,** Kremnické vrchy Mts.	Badín village, ca. 5 km NW of the church, the National Nature Reserve Badínsky prales	old growth forest of *Fagus sylvatica*, *Abies alba*	on wood of fallen trunk of *Fagus sylvatica*, 4th decay stage	48°41'12"N, 19°03'07"E	750	9.IX.2015, S. Jančovičová
	SLO 1826*	**Slovakia,** Muránska planina Plateau	Tisovec town, W of the ground elevation Hradová, the National Nature Reserve Hradová	forest of *Fagus sylvatica*	on wood of fallen trunk of *Fagus sylvatica*, 4th decay stage	48°40'50.1"N 19°55'16.3"E	850	26.VI.2008, D. Blanár
	SLO 2548*	**Slovakia,** Muránska planina Plateau	Muráň village, ca. 3 km NNW of the church, the National Nature Reserve Poludnica	forest of *Fagus sylvatica*	on wood of fallen trunk of *Fagus sylvatica*, 4th decay stage	48°45'47"N, 20°02'35"E	550	21.VI.2002, D. Blanár
	SLO 479*	**Slovakia,** Muránska planina Plateau	Muráň village, ca. 3.5 km W of the church, the National Nature Reserve Javorníková	forest of *Fagus sylvatica*, *Abies alba*	on wood of fallen trunk of *Fagus sylvatica*, 4th decay stage	48°44'15"N, 20°00'19"E	500	27.X.2001, S. Jančovičová
	SLO 2091*	**Slovakia,** Slanské vrchy Mts.	Rankovce village, ca. 3 km E of the church, the Nature Reserve Rankovské skaly	forest of *Fagus sylvatica*, *Fraxinus*, *Quercus*	on wood of standing trunk of *Fraxinus excelsior*, 4th decay stage	48°49'29.8"N, 21°29'39.0"E	670	8.VII.2001, S. Jančovičová
	SLO 2536	**Slovakia,** Vihorlatské vrchy Mts.	Remetské Hámre village, ca. 8 km N of the church, the Nature Reserve Jedlinka	forest of *Fagus sylvatica*, *Abies alba*	on wood of fallen trunk of *Fagus sylvatica*, 4th decay stage	48°54'51"N, 22°10'49"E	800	19.IX.2003, S. Jančovičová
	SLO 2540*	**Slovakia,** Vihorlatské vrchy Mts.	Remetské Hámre village, ca. 8 km N of the church, the Nature Reserve Jedlinka	forest of *Fagus sylvatica*, *Abies alba*	on wood of fallen trunk of *Fagus sylvatica*, 4th decay stage	48°54'51"N, 22°10'49"E	800	10.VI.2003, S. Jančovičová
	SLO 2546	**Slovakia,** Vihorlatské vrchy Mts.	Remetské Hámre village, ca. 1 km E of the church, the Nature Reserve Machnatý vrch	acidophilous *Fagus sylvatica* forest	on wood of fallen trunk of *Fagus sylvatica*, 4th decay stage	48°50'54"N 22°11'24"E	300–350	12.VII.2001, J. Ripka
	SLO 2544*	**Slovakia,** Vihorlatské vrchy Mts.	Remetské Hámre village, ca. 1 km E of the church, the Nature Reserve Machnatý vrch	acidophilous *Fagus sylvatica* forest	on wood of fallen trunk of *Fagus sylvatica*, 4th decay stage	48°50'54"N 22°11'24"E	300–350	12.VII.2001, S. Jančovičová
	SLO 2542	**Slovakia,**	Remetské Hámre village, ca. 1 km	acidophilous *Fagus sylvatica*	on wood of fallen trunk of	48°50'54"N	300–350	20.IX.2003,

		Vihorlatské vrchy Mts.	E of the church, the Nature Reserve Machnatý vrch	forest	*Fagus sylvatica*, 4th decay stage	22°11'24"E		V. Kučera
	SLO 2530*	**Slovakia,** Vihorlatské vrchy Mts.	Remetské Hámre village, ca. 8 km NNE of the church, the National Nature Reserve Morské oko	forest of *Fagus sylvatica*, *Abies alba*	on wood of fallen trunk of *Fagus sylvatica*, 4th decay stage	48°54'54"N, 22°12'06"E	650	19.IX.2003, S. Jančovičová
	SLO 2526	**Slovakia,** Vihorlatské vrchy Mts.	Remetské Hámre village, ca. 8 km NNE of the church, the National Nature Reserve Morské oko	forest of *Fagus sylvatica*, *Abies alba*	on wood of fallen trunk of deciduous tree, 4th decay stage	48°54'54"N, 22°12'06"E	650	14.VIII.2000, S. Jančovičová
	SLO 2527	**Slovakia,** Vihorlatské vrchy Mts.	Remetské Hámre village, ca. 8 km NNE of the church, the National Nature Reserve Morské oko	forest of *Fagus sylvatica*, *Abies alba*	on wood of fallen trunk of *Fagus sylvatica*, 4th decay stage	48°54'54"N, 22°12'06"E	650	12.VII.2001, S. Jančovičová
	SLO 2529	**Slovakia,** Vihorlatské vrchy Mts.	Remetské Hámre village, ca. 8 km NNE of the church, the National Nature Reserve Morské oko	forest of *Fagus sylvatica*, *Abies alba*	on wood of stump of *Fagus sylvatica*, 4th decay stage	48°54'54"N, 22°12'06"E	650	12.VII.2001, S. Jančovičová
	SLO 2518	**Slovakia,** Vihorlatské vrchy Mts.	Hlivištia village, ca. 1.5 km E of the church	forest of *Carpinus betulus*, *Quercus robur*, *Q. petraea*, *Fagus sylvatica*	on wood of fallen trunk of *Fagus sylvatica*, 4th decay stage	48°48'19"N, 22°13'53"E	280	13.VII.2001, S. Jančovičová
	SLO 2519	**Slovakia,** Vihorlatské vrchy Mts.	Hlivištia village, ca. 1.5 km E of the church	forest of *Carpinus betulus*, *Quercus robur*, *Q. petraea*, *Fagus sylvatica*	on wood of stump of *Fagus sylvatica*, 4th decay stage	48°48'19"N, 22°13'53"E	280	13.VII.2001, J. Terray
	SLO 2520	**Slovakia,** Vihorlatské vrchy Mts.	Hlivištia village, ca. 1.5 km E of the church	forest of *Carpinus betulus*, *Quercus robur*, *Q. petraea*, *Fagus sylvatica*	on wood of fallen trunk of *Fagus sylvatica*, 4th decay stage	48°48'19"N, 22°13'53"E	280	13.VII.2001, J. Terray
	SLO 2541*	**Slovakia,** Bukovské vrchy Mts.	ca. 2 km NNE of the former village of Ruské	forest of *Fagus sylvatica* and mixed beech forest	on wood of fallen branch of *Fagus sylvatica*, 4th decay stage	49°07'38"N, 22°20'33"E	550	23.VII.2020, S. Jančovičová
	SLO 1845*	**Russia,** the Republic of Tatarstan	Raifa village, ca. 3 km SSW of the churche, the Volzhsko-Kamsky National Nature Biosphere Reserve	forest of *Tilia*, *Acer*, *Quercus*, *Populus tremula*	on wood of fallen trunk of *Quercus*, 4th decay stage	55°52'28.3"N, 48°43'7.6"E	90	23.VIII.2016, T. Svetasheva
	SLO 2394*	**Czech Republic,** Drahanská vrchovina Upland	Brno city, ca. 0.5 km NE of the borough of Brno-Útěchov, the Nature Reserve Coufavá	forest of *Fagus sylvatica*, *Quercus petraea*, *Carpinus betulus*, *Abies alba*	on wood of fallen trunk of *Fagus sylvatica*, 4th decay stage	49°17'39.7"N, 16°38'15.3"E	440	30.VIII.2017, D. Dvořák
	SLO 2549	**Czech Republic,** Drahanská vrchovina Upland	Brno city, ca. 0.5 km NE of the borough of Brno-Útěchov, the Nature Reserve Coufavá	forest of *Fagus sylvatica*, *Quercus petraea*, *Carpinus betulus*, *Abies alba*	on wood of fallen trunk of *Fagus sylvatica*, 4th decay stage	49°17'39.7"N, 16°38'15.3"E	440	30.VIII.2017, D. Dvořák
Crepidotus pini	SLO 2550*	**Slovakia,** Záhorská nížina Lowland	Záhorie military district, Malacky town, ca. 4 km E of the hospital, game preserve	about 80 years old forest of *Pinus sylvestris* with admixed *Quercus*	on wood of stump of *Pinus sylvestris*, 4th decay stage	48°26'15"N, 17°05'03"E	190	30.IX.2017, S. Jančovičová
	SLO 2601*	**Slovakia,** Záhorská nížina Lowland	Záhorie military district, Rohožník village, ca. 3 km W of the church	about 100 years old forest of *Pinus sylvestris*	on wood of stump of *Pinus sylvestris*, 3rd decay stage	48°27'18"N, 17°07'39"E	220	28.VIII.2021, S. Jančovičová
	SLO 2602	**Slovakia,** Záhorská nížina	Záhorie military district, Rohožník village, ca. 3 km W of the church	about 100 years old forest of *Pinus sylvestris*	on wood of stump of *Pinus sylvestris*, 3rd decay stage	48°27'18"N, 17°07'39"E	220	19.IX.2021, S. Jančovičová

		Lowland						
	SLO 2579*	**Slovakia,** Záhorská nížina Lowland	Záhorie military district, Rohožník village, ca. 1.5 km W of the church	about 100 years old forest of *Pinus sylvestris* and self-sown *Carpinus betulus*, *Quercus* and *Crataegus*	on wood of stump of *Pinus sylvestris*, 4th decay stage	48°27'41"N, 17°08'52"E	210	12.IX.2020, S. Jančovičová
	SLO 2623	**Slovakia,** Záhorská nížina Lowland	Záhorie military district, Rohožník village, ca. 1.5 km W of the church	about 100 years old forest of *Pinus sylvestris* and self-sown *Carpinus betulus*, *Quercus* and *Crataegus*	on wood of stump of *Pinus sylvestris*, 4th to 5th decay stage	48°27'41"N, 17°08'52"E	210	26.IX.2021, S. Jančovičová
	SLO 2622	**Slovakia,** Záhorská nížina Lowland	Záhorie military district, Rohožník village, ca. 1.5 km W of the church	about 100 years old forest of *Pinus sylvestris* and self-sown *Carpinus betulus*, *Quercus* and *Crataegus*	on wood of stump of *Pinus sylvestris*, 4th decay stage	48°27'41"N, 17°08'52"E	210	10.X.2021, S. Jančovičová
Crepidotus stenocystis	SLO 2557*	**Slovakia,** Liptovská kotlina Basin	Važec village, ca. 4 km NNE of the church, the forest along the Solisková voda creek	forest of *Picea abies*, *Betula*, *Salix*	on wood of fallen trunk of *Abies alba*, 4th decay stage	49°05'36"N, 19°59'59"E	880	29.IX.2003, I. Kautmanová
	SLO 481*	**Slovakia,** Veporské vrchy Mts.	Polomka village, ca. 8.5 km SSE of the church, the Nature Reserve Fabova hoľa	forest of *Picea abies* with *Sorbus aucuparia* in the understory	on wood of stump of *Picea abies*, 4th decay stage	48°46'47.1"N, 19°52'51"E	1360	1.X.2009, D. Blanár
