# Supplementary material for: Phylogeny of Crepidotus applanatus Look-Alikes Reveals a Convergent Morphology Evolution and a New Species C. pini
Source: J Fungi (Basel). 2022 May 7;8(5):489. doi: 10.3390/jof8050489 (PMC9144983; doi:10.3390/jof8050489)
Supplement: Supplementary file 1 [file jof-08-00489-s001.zip › Table S2.pdf]

Table S2: ITS and LSU nrDNA sequences of *Crepidotus* and of the outgroup used in the phylogenetic analysis. Sequences obtained in this study are in bold. Full citations of published sources are bellow.

Taxon	Specimen voucher / isolate	Country of origin	GenBank no. ITS	GenBank no. LSU	Sources
C. aureus	OKM27300	Puerto Rico	-	AF205685	Aime (1999)
C. alabamensis	MCA778	USA	-	AF367960	Aime, M.C. (unpubl.)
C. albolanatus	PDD 72865 (holotype)	New Zealand	NR159820	-	Horak (2018)
C. amygdalosporus	OKM 26740	USA (Virginia)	-	AF205678	Aime (1999)
	MCA258	USA (Virginia)	-	AF205675	Aime (1999)
C. antilarum	OKM26827	Dominican Republic	-	AF205680	Aime (1999)
C. applanatus	SLO 2551	Slovakia	**OM832526**	**OM832560**	**This study**
	SLO 2534	Slovakia	**OM832521**	**OM832556**	**This study**
	SLO 2537	Slovakia	**OM832522**	**OM832557**	**This study**
	SLO 2539	Slovakia	**OM832523**	**OM832558**	**This study**
	SLO 2533	Slovakia	**OM832524**	**OM832555**	**This study**
	SLO 2528	Slovakia	**OM832520**	**OM832554**	**This study**
	SLO 2547	Slovakia	**OM832525**	**OM832559**	**This study**
	SLO 2581	Norway	**OM832528**	**OM832561**	**This study**
	WU 29809	Germany	KF879613	-	Jančovičová et al. (2014)
	WU 29794	Austria	KF879614	-	Jančovičová et al. (2014)
	SLO 1821	Slovakia	MF621028	-	Jančovičová et al. (2017)
	SLO 1492	Slovakia	MF621029	-	Jančovičová et al. (2017)
	KA12-0350	Republic of Korea	KR673425	-	Kim et al. (2015)
	KA12-0390	Republic of Korea	KR673447	-	Kim et al. (2015)
	KA12-1370	Republic of Korea	KR673581	-	Kim et al. (2015)
	KA13-0932	Republic of Korea	KR673659	-	Kim et al. (2015)
	BIOUG24046-F04	Canada (Ontario)	KT695357	-	Telfer (2015)
	TENN 61392 clone C3	USA (Tennessee)	FJ596803	-	Hughes et al. (2009)
	NAMA 2015-353 Mushroom Observer # 247314	USA (North Carolina)	MH910575	-	Russell, S.D.; Rojas, J.A.; Vilgalys, R. (unpubl.)
	D93-F12-ITS4_K23.ab1	USA (Arkansas)	MN826231	-	Alshammari, N.I.; Stephenson, S.L. (unpubl.)
	D93-F6-ITS1_K11.ab1	USA (Arkansas)	MN826230	-	Alshammari, N.I.; Stephenson, S.L. (unpubl.)
	MHHNU 31061	China	MK250928	-	Chen, Z.H.; Zhang, P.; Li, S.N. (unpubl.)
	PUL F27036	USA (Wisconsin)	MW448624	-	Dirks, A.C. (unpubl.)
	180524-H06	Republic of Korea	MN082534	-	Cho, J.-H.; An, G.-H.; Han, J.-G. (unpubl.)
	420526MF0689	China	-	MG712368	Wang, R.; Zhang, X., Yan, S.Z.;

					Chen, S.L. (unpubl.)
	420526MF0534	China	-	MH141341	Wang, R.; Zhang, X., Yan, S.Z.; Chen, S.L. (unpubl.)
	TM03_415	Canada	-	EU522804	Porter et al. (2008)
	MCA 170	USA (Virginia)	-	AF205694	Aime (1999)
C. applanatus var. *globigera*	MCA 188	USA (Virginia)	-	AF205673	Aime (1999)
C. asiaticus	TJB9995LSU (holotype)	Thailand	-	MF077336	Guzmán-Dávalos et al. (2017)
C. cf. *applanatus*	PBM717	USA (Washington)	DQ202273	AY380406	Matheny (2007) / Matheny (2005)
	Montri-173	Switzerland	MK028392	-	Hofstetter et al. (2019)
	WTU-F-073211	USA (Washington)	MT946689	-	Trudell, S.; Gordon, M. (unpubl.)
C, betulae	MCA384	USA (North Carolina)		AF205679	Aime (1999)
C. brunnescens	MICH10455 (paratype)	USA (Michigan)	-	MF621025	Jančovičová et al. (2017)
	MCA 864	USA (Virginia)	-	AF367936	Aime (2001)
C. calolepis	TUF118750	Estonia	UDB019553		https://unite.ut.ee
C. cesatii	DB3782	Hungary	-	MK277881	Varga et al. (2019)
	TUF120093	Estonia	UDB023726	-	https://unite.ut.ee
C. cinnabarinus	MCA387	USA (xxx)	-	AF205686	Aime (1999)
C. crocophyllus	SLO 2588	Slovakia	**OM832530**	**OM832563**	**This study**
	SLO 2433	Slovakia	**OM832529**	**OM832562**	**This study**
	SLO 2590	Slovakia	**OM832531**	**OM832564**	**This study**
	SJ1	Slovakia	-	AF367939	Aime (2001)
	KA12-0349	Republic of Korea	KR673424	-	Kim et al. (2015)
	KA12-1376	Republic of Korea	KR673586	-	Kim et al. (2015)
	KA12-1407	Republic of Korea	KR673601	-	Kim et al. (2015)
	SLO 2553	USA (North Carolina)	**OM832527**	-	**This study**
	TENN 61519 clone C1	USA (Tennessee)	FJ596821	-	Hughes et al. (2009)
	PBM3047	USA (California)	-	GQ893025	Kumar et al. (2020)
	OKM 26173	USA (Missouri)	-	AY029707	Aime (2001)
	OKM 25806	USA (Virginia)	-	AF367938	Aime (2001)
	RGT 930924/02	USA (Wisconsin)	-	AF139946	Aime (2001)
C. dentatus	HMJAU37097 (holotype)	China	NR173279		Ge & Bau (2020)
C. distortus	MCA 386	USA (Virginia)	-	AF205671	Aime (1999)
C. ehrendorferi	SLO 2558	Slovakia	**OM832533**	-	**This study**
	SLO 2587	Slovakia	**OM832534**	**OM832566**	**This study**
	SLO 707	Slovakia	**OM832532**	**OM832565**	**This study**
	WU 6554 (holotype)	Austria	MH780918	-	Liu et al. (2018)
	WU 20080	France	MH780919	-	Liu et al. (2018)
	PV 218	Hungary	MH780920	-	Liu et al. (2018)
C. ellipsoideus	G1955	USA	-	MK277883	Varga et al. (2019)
C. epibryus	TUF118984	Estonia	UDB023702	-	https://unite.ut.ee
	IBNR1997/0948	Russia	-	AF367934	Aime et al. (2005)

C. exiguus	TBGT17176 (holotype)	India	-	MK567974	Kumar et al. (2022)
C. exilis	TBGT17157 (holotype)	India	-	MK878548	Kumar et al. (2020)
C. fragilis	MCA904	USA (Virginia)	-	AF367931	Aime et al. (2005)
C. fraxinicola	OKM26739	USA (Washington)	-	AF205676	Aime (1999)
C. globisporus	TBGT17341 (holotype)	India	-	MK878540	Kumar et al. (2020)
C. indicus	TBGT17161 (holotype)	India	-	MG735357	Kumar et al. (2018a)
C. inhonestus	MCA638	Japan	-	AF205704	Moncalvo et al. (2002)
C. iqbalii	LAH 36654 (holotype)	Pakistan	NR173306	NG079698	Izhar et al. (2021)
C. kauffmanii	G1956	USA	-	MK277887	Varga et al. (2019)
C. lanuginosus	OKM27331	USA (Idaho)	-	AF367940	Aime & Miller (2002)
C. lateralipes	PDD 72508 (holotype)	New Zealand	NR159821	-	Horak (2018)
C. lundelii	TUF106886	Estonia	UDB015199	-	https://unite.ut.ee
	IBNR1997/0946	Russia	-	AF367941	Aime, M.C. (unpubl.)
C. luteolus	16834	Italy	JF907963	-	https://nature.berkeley.edu
C. lutescens	HMJAU 37002 (holotype)	China	NR158400	-	Ge et al. (2017)
C. macedonicus	PV773	Hungary	MH780921	-	Liu et al. (2018)
C. malachioides	SLO 2578	Slovakia	**OM832538**	**OM832568**	**This study**
	SLO 2391	Slovakia	**OM832536**	**OM832567**	**This study**
	SLO 2561	Slovakia	**OM832537**	-	**This study**
	SLO 1932	Slovakia	**OM832535**	-	**This study**
	SLO 1250 (epitype)	Slovakia	KF154019	KF154018	Jančovičová et al. (2014)
	WU 31421	Austria	KF879616	-	Jančovičová et al. (2014)
	WU 32709	Austria	KF879615	-	Jančovičová et al. (2014)
C. malachius	SLO 2552	Slovakia	**OM832548**	**OM832578**	**This study**
	SLO 1826	Slovakia	**OM832539**	**OM832569**	**This study**
	SLO 2548	Slovakia	**OM832544**	**OM832577**	**This study**
	SLO 2091	Slovakia	**OM832541**	**OM832571**	**This study**
	SLO 2540	Slovakia	**OM832545**	**OM832574**	**This study**
	SLO 2544	Slovakia	**OM832547**	**OM832576**	**This study**
	SLO 2530	Slovakia	**OM832543**	**OM832573**	**This study**
	SLO 2541	Slovakia	**OM832546**	**OM832575**	**This study**
	SLO 1845	Russia (Republic of Tatarstan)	**OM832540**	**OM832570**	**This study**
	SLO 2394	Czech Republic	**OM832542**	**OM832572**	**This study**
	SLO 1497	Slovakia	MF621032	-	Jančovičová et al. (2017)
	SLO 479	Slovakia	MF621033	-	Jančovičová et al. (2017)
	WU 29795	Austria	KF879618	-	Jančovičová et al. (2014)
	IBL16	Poland	MZ410645	-	Rosa-Gruszecka, A.; Hilszczanska, D.; Haidt, A.; Tereba, A.; Wrobel, M.; Stachyra, P. (unpubl.)
C. malachius var. *malachius*	MCA 343	USA (Virginia)	-	AF205674	Aime (1999)

	MCA 775	USA (North Carolina)	-	AY029706	Aime (2004)
C. malachius var. *trichifer*	MCA 719	Japan	-	AF367943	Aime (2001)
C. martini	MCA640	Japan	-	AF367944	Aime, M.C. (unpubl.)
C. melleus	MCA672	Japan	-	AF205702	Aime (1999)
C. mollis	DMS-9344564	Denmark	MT640263	MT640263	Leerhoei,F. and Langkjaer,E.M. (unpubl.)
	TUF120313	Estonia	UDB024710	-	https://unite.ut.ee
	NL-3003	Hungary	-	MK277890	Varga et al. (2019)
C. nephrodes	OKM 25896	USA (Virginia)	-	AF205693	Aime (1999)
	MCA 189	USA (Virginia)	-	AF205670	Aime (1999)
C. nyssicola	TJB 8699	USA (Maine)	-	AF205690	Aime (1999)
C. palodensis	TBGT16716 (holotype)	India	-	MH310743	Kumar et al. (2018a)
C. pini	SLO 2579	Slovakia	**OM832550**	**OM832580**	**This study**
	SLO 2550	Slovakia	**OM832549**	**OM832579**	**This study**
	SLO 2601	Slovakia	**OM832551**	-	**This study**
C. podocarpi	OKM27303	Puerto Rico	-	AF205696	Aime (1999)
C. pseudomollis	HMJAU37125 (holotype)	China	NR173280	-	Ge & Bau (2020)
C. roseus	TBGT15507	India	-	MK567977	Kumar et al. (2022)
C. rufidulus	PDD 98272 (isotype)	New Zealand	NR159823	-	Horak (2018)
C. rufofloccosus	PDD 72601 (holotype)	New Zealand	NR159822	-	Horak (2018)
C. sinuosus	OKM26290	USA	-	AF367954	Aime, M.C. (unpubl.)
Crepidotus sp.	MCA 679	Japan	-	AF367952	Aime (2001)
	MCA 941	Japan	-	AF367954	Aime (2001)
	TNS B225	Japan	KF680278	-	Kasuya et al. (2014)
	TBGT15841	India	-	MK567981	Kumar, A.M.; Pradeep, C.K.; Vrinda, K.B. (unpubl.)
	MCA 442	Puerto Rico	-	AF367950	Aime (2004)
C. sphaerosporus	OKM27013	USA (Colorado)	-	AF205682	Aime & Miller (2002)
C. stenocystis	SLO 2557	Slovakia	**OM832553**	**OM832581**	**This study**
	SLO 481	Slovakia	**OM832552**	-	**This study**
	PRM 911279 (holotype)	Czech Republic	MF621030	MF621024	Jančovičová et al. (2017)
C. striatus	HMJAU37076 (holotype)	China	NR173281	-	Ge & Bau (2020)
C. subverrucisporus	O-F-248331	Norway	UDB037991	-	https://unite.ut.ee
C. tennesseensis	TENN:029144 (holotype)	USA (Tennessee)	NR119720	-	Schoch et al. (2014)
C. thermophilus	TJB8496	Puerto Rico	-	AF205691	Aime et al. (2002)
C. tobolensis	LE287655 (holotype)	Russia	NR174894	MK560762	Crous et al. (2019)
C. tortus	TBGT17194 (holotype)	India	-	MK462162	Kumar et al. (2022)
C. trichocraspedotus	HMJAU37138 (holotype)	China	NR173282	-	Ge & Bau (2020)
C. uber	MCA1403	USA	-	AF367961	Aime, M.C. (unpubl.)
C. variabilis	SLO 2018	Slovakia	MT055890	**OM832583**	Jančovičová et al. (2020) / **This study**
	SLO 2021	Slovakia	MT055889	**OM832585**	Jančovičová et al. (2020) / **This**

					**study**
	SLO 2016	Slovakia	MT055888	**OM832582**	Jančovičová et al. (2020) **/ This study**
	SLO 2423	Slovakia	MT055887	**OM832584**	Jančovičová et al. (2020) **/ This study**
C. versutus	IBNR1997/0962	Russia	-	AF367958	Aime, M.C. (unpubl.)
C. volubilis	TBGT15648 (holotype)	India	-	MH310742	Kumar et al. (2018b)
Simocybe phlebophora	PBM3089	New Zealand	MK421963	MK421967	Matheny et al. (2020)
Simocybe rhabarbarina	4583-15	Italy	KT934412	KT934415	Poli et al. (2015)
Simocybe sp.	PBM3031	USA (Tennessee)	GQ893023	GQ892979	Matheny et al. (2020)

References to Supplementary Table S2

Aime, M.C. Generic concepts in the Crepidotaceae as inferred from nuclear large subunit, ribosomal DNA sequences, morphology, and basidiospore dormancy patterns. MSc Thesis, Virginia Polytechnic Institute and State University, Blacksburg, USA (Virginia), 1999.

Aime, M.C. Biosystematic Studies in *Crepidotus* and the Crepidotaceae (Basidiomycetes, Agaricales). PhD Thesis, Virginia Polytechnic Institute and State University, Blacksburg, USA (Virginia), 2001.

Aime, M.C. Intercompatibility tests and phylogenetic analysis in the *Crepidotus Sphaerula* group complex: concordance between ICGs and nuclear rDNA sequences highlight phenotypic plasticity within Appalachian species. In Fungi in forest ecosystems: systematics, diversity, and ecology; Cripps, C.L. Ed.; New York Botanical Garden: New York, USA, 2004; pp. 71–92.

Aime, M.C.; Baroni, T.J.; Miller, O.K. Crepidotus thermophilus comb. nov., a reassessment of *Melanomphalia thermophila*, a rarely collected tropical agaric. Mycologia 2002, 94,1059–1065.

Aime, M,C.; Miller, O.K. Delayed germination of basidiospores in temperate species of *Crepidotus* (Fr.) Staude. Can. J. Bot. 2002, 80, 280–287.

Aime, M.C.; Vilgalys, R.; Miller, O.K. The Crepidotaceae (Basidiomycota, Agaricales): phylogeny and taxonomy of the genera and revision of the family based on molecular evidence. Am. J. Bot. 2005, 92, 74–82.

Crous, P.W. et al. Fungal Planet description sheets: 868–950. Persoonia 2019, 42, 291–473

Ge, Y.P.; Bau, T. Descriptions of six new species of *Crepidotus* from China. Mycosystema. 2020, 39, 238–255.

Ge, Y.P.; Yang, S.S.; Bau, T. *Crepidotus lutescens* sp. nov. (Inocybaceae, Agaricales), an ochraceous salmon colored species from northeast of China. Phytotaxa 2017, 297, 189–196.

Guzmán-Dávalos L.; Pradeep C.K.; Vrinda K.B.; Kumar A.M.; RamírezCruz V.; Herrera M.; Villalobos-Arámbula A.R.; Soytong K.; Baroni T.J.; Aime M.C. A new stipitate species of *Crepidotus* from India and Thailand, with notes on other tropical species. Mycologia 2017,109, 804–814.

Hofstetter, V.; Buyck, B.; Eyssartier, G.; Schnee, S.; Gindro, K. The unbearable lightness of sequenced-based identification. Fungal Diversity 2019, 96, 243–284.

Horak, E. Fungi of New Zealand. Volume 6. Agaricales (Basidiomycota) of New Zealand. 2. Brown spored genera p.p. *Crepidotus*, *Flammulaster*, *Inocybe*, *Phaeocollybia*, *Phaeomarasmius*, *Pleuroflammula*, *Pyrrhoglossum*, *Simocybe*, *Tubaria* and *Tympanella*. Westerdijk Biodiversity Series 2018, 16, 1–205.

Hughes, K.W.; Petersen, R.H.; Lickey, E.B. Using heterozygosity to estimate a percentage DNA sequence similarity for environmental species' delimitation across basidiomycete fungi. New Phytologist 2009, 182: 795–798.

Izhar, A.; Usman, M.; Khalid, A.N. *Crepidotus iqbalii* (Crepidotaceae, Agaricales) a new stipitate species, from Pakistan. Phytotaxa 2021, 500, 95–107.

Jančovičová, S.; Tomšovský, M.; Urban, A.; Krisai-Greilhuber, I. New records and epitypification of *Crepidotus malachioides* (Crepidotaceae, Agaricales). Sydowia 2014, 66, 79–97.

Jančovičová, S.; Adamčík, S.; Looney, B.P.; Caboň, M.; Čaplovičová, M.; Kopáni, M.; Pennycook, S.R.; Adamčíková, K. Delimitation of European *Crepidotus stenocystis* as different from the North American species *C. brunnescens* (Crepidotaceae, Agaricales). Phytotaxa 2017, 328, 127–139.

Jančovičová, S.; Adamčíková, K.; Caboň, M.; Adamčík, S. How variable is *Crepidotus variabilis*? Phytotaxa 2020, 449, 253–264.

Kasuya, T.; Uno, K.; Hosaka K. Reexamination of *Crepidotus crocophyllus* (Basidiomycota, Fungi) in Japan, with reference to its phylogenetic placement. Univ. Bull. Chiba Inst. Sci 2014, 7: 159–166.

Kim, C.S.; Jo, J.W.; Kwag, Y.N.; Sung, G.H.; Lee, S.G.; Kim, S.Y.; Shin, C.H.; Han, S.K. Mushroom flora of Ulleung-gun and a newly recorded *Bovista* species in the Republic of Korea. Mycobiology 2015, 43: 239–257.

Kumar, A.M,; Vrinda, K.B.; Pradeep, C.K. New and noteworthy crepidotoid agarics from India. Cryptogam., Mycol 2018a 39, 287–298.

Kumar, A,M.; Vrinda, K.B.; Pradeep, C.K. Two new species of *Crepidotus* (Basidiomycota, Agaricales) from peninsular India. Phytotaxa 2018b, 372, 67–78

Kumar, A.M.; Aime, M.C.; Vrinda, K.B.; Pradeep, C.K. Two new species and a new record of *Crepidotus* (Agaricomycetes) from India. Aust. Systematic Bot. 2020, 33, 380–391.

Kumar, A.M.; Pradeep, C.K.; Aime, M.C. New species and new records of *Crepidotus* (Crepidotaceae) from India. Mycol. Prog. 2022, 21, 311–326.

Liu L.-N., Razaq A., Atri N.S., Bau T., Belbahri L., Chenari Bouket A., Chen L.-P., Deng C., Ilyas S., Khalid A.N., Kitaura M.J., Kobayashi T., Li Y., Lorenz A.P., Ma Y.-H., Malysheva E., Malysheva V., Nuytinck J., Qiao M., Saini M.K., Scur M.C., Sharma S., Shu L.-L., Spirin V., Tanaka Y., Tojo M., Uzuhashi S., Valério-Júnior C., Verbeken A., Verma B., Wu R.-H., Xu J.-P., Yu Z.-F., Zeng H., Zhang B., Banerjee A., Beddiar A., Bordallo J.J., Dafri A., Dima B., Krisai-Greilhuber I., Lorenzini M., Mandal R., Morte A., Nath P.S., Papp V., Pavlík J., Rodríguez A., Ševcˇíková H., Urban A., Voglmayr H. & Zapparoli G. (2018) Fungal Systematics and Evolution: FUSE 4. – Sydowia 70: 211–286.

Matheny, P.B. et al. Contributions of rpb2 and tef1 to the phylogeny of mushrooms and allies (Basidiomycota, Fungi). Mol. Phylogenet. Evol. 2007, 43, 430–451.

Matheny, P.B.; Hobbsa A.M.; Esteve-Raventós, F. Genera of Inocybaceae: New skin for the old ceremony. Mycologia 2020, 112: 83–120.

Moncalvo, J.-M.; Vilgalys, R.; Redhead, S.A.; Johnson, J.E.; James T.Y.; Aime, M.C.; Hofstetter, V.; Verduin, S.J.W.; Larsson, E.; Baroni, T.J.; Thorn, G.; Jacobsson, S.; Clémençon, H.; Miller, O.K. Jr. One hundred and seventeen clades of euagarics. Mol. Phylogenet. Evol. 2002, 23: 357–400.

Poli, L.; Musumeci, E.; Alvarado, P. A new European *Simocybe* discovered in (Brianza) Lombardia: *S. rhabarbarina* sp. nov. Boll Assoc Micol Ecol Romana 2015, 96: 20–30.

Porter, T.M.; Skillman, J.E.; Moncalvo, J.M. Fruiting body and soil rDNA sampling detects complementary assemblage of Agaricomycotina (Basidiomycota, Fungi) in a hemlock-dominated forest plot in southern Ontario. Mol. Ecol. 2008, 17, 3037–3050.

Schoch, C.L et al. 2014. Finding needles in haystacks: linking scientific names, reference specimens and molecular data for Fungi. Database 2014, bau061, 21 p.

Telfer et al. Biodiversity inventories in high gear: DNA barcoding facilitates a rapid biotic survey of a temperate nature reserve. Biodiversity Data Journal 2015, 3, e6313.

Varga et al. Megaphylogeny resolves global patterns of mushroom evolution. Nat Ecol Evol 2019, 3, 668–678.
