# Supplementary material for: Phylogeny of Crepidotus applanatus Look-Alikes Reveals a Convergent Morphology Evolution and a New Species C. pini
Source: J Fungi (Basel). 2022 May 7;8(5):489. doi: 10.3390/jof8050489 (PMC9144983; doi:10.3390/jof8050489)
Supplement: Supplementary file 1 [file jof-08-00489-s001.zip › Table S3.pdf]

Table S3: Estimated ranges of basidiospore dimensions of the studied *Crepidotus* collections based on 30 measurements represented as average (underlined) ± standard deviation, in parenthesis minimum and maximum values, Q = length/width ratio. Sequenced specimens are marked with asterisk.

**Taxon**	**Collection number**	**Length (µm)**	**Width (µm)**	**Q**
Crepidotus applanatus	SLO 1492*	(4.8)4.9–5.2–5.5(6.2)	(4.5)4.8–5.1–5.5(6.2)	0.99–1.01–1.03
	SLO 2551*	(4.8)4.8–5.2–5.7(6.8)	(4.8)4.8–5.2–5.6(6.8)	0.99–1.00–1.02
	SLO 2517	(4.8)4.8–5.0–5.2(5.8)	(4.8)4.8–5.0–5.3(5.8)	0.98–1.00–1.02
	SLO 1821*	(4.8)4.9–5.1–5.3(5.7)	(4.8)4.9–5.1–5.3(5.7)	0.98–1.00–1.03
	SLO 2524	(5.0)4.9–5.3–5.6(6.0)	(5.0)4.9–5.2–5.6(6.0)	0.99–1.00–1.02
	SLO 2523	(4.5)4.7–5.1–5.5(6.5)	(4.5)4.7–5.1–5.4(6.5)	0.99–1.00–1.02
	SLO 2534*	(4.8)4.8–5.1–5.4(6.0)	(4.8)4.8–5.1–5.4(6.0)	0.98–1.01–1.03
	SLO 2535	(4.8)5.0–5.2–5.5(6.0)	(4.8)5.0–5.2–5.5(6.0)	0.99–1.00–1.01
	SLO 2537*	(4.5)4.8–5.1–5.4(6.0)	(4.5)4.8–5.1–5.3(5.8)	0.99–1.00–1.02
	SLO 2538	(4.8)4.9–5.2–5.4(6.0)	(4.8)4.9–5.1–5.4(6.0)	0.99–1.00–1.02
	SLO 2539*	(4.5)4.8–5.2–5.5(6.0)	(4.5)4.8–5.2–5.5(6.0)	0.98–1.00–1.02
	SLO 2533*	(4.8)4.9–5.2–5.4(5.8)	(4.8)4.9–5.1–5.4(5.8)	0.99–1.00–1.02
	SLO 2545	(4.5)4.8–5.2–5.7(6.2)	(4.5)4.8–5.2–5.7(6.2)	0.99–1.00–1.02
	SLO 2525	(5.0)5.0–5.4–5.8(6.2)	(4.8)4.9–5.3–5.8(6.2)	0.99–1.00–1.02
	SLO 2528*	(4.2)4.6–5.0–5.4(5.8)	(4.2)4.6–5.0–5.4(5.8)	0.99–1.00–1.06
	SLO 2531	(4.5)4.7–5.0–5.2(5.5)	(4.5)4.6–5.0–5.2(5.5)	0.98–1.01–1.03
	SLO 2532	(4.5)4.8–5.1–5.4(6.0)	(4.5)4.8–5.1–5.4(5.8)	0.99–1.00–1.02
	SLO 2547*	(4.8)5.0–5.3–5.6(6.0)	(4.8)5.0–5.3–5.5(6.0)	0.99–1.00–1.01
	SLO 1926	(4.8)4.9–5.4–5.9(6.2)	(4.8)4.9–5.3–5.8(6.0)	0.99–1.02–1.05
	SLO 2581*	(4.5)4.9–5.4–5.9(6.8)	(4.5)4.9–5.3–5.8(6.8)	0.99–1.00–1.02
Crepidotus malachius	SLO 2552*	(5.2)6.2–6.8–7.4(8.0)	(5.2)6.1–6.7–7.3(8.0)	0.99–1.01–1.02
	SLO 1497*	(5.2)6.0–6.7–7.4(8.0)	(5.2)6.0–6.6–7.3(8.0)	0.99–1.01–1.01
	SLO 1826*	(5.8)5.9–6.5–7.1(9.0)	(5.8)5.9–6.5–7.1(9.0)	0.99–1.00–1.01
	SLO 2548*	(5.8)6.4–7.1–7.7(9.0)	(5.8)6.3–7.1–7.8(9.0)	0.98–1.00–1.02
	SLO 479*	(5.2)5.6–6.6–7.6(9.5)	(5.2)5.6–6.6–7.7(9.5)	0.99–1.00–1.01
	SLO 2091*	(5.5)5.9–6.6–7.3(8.2)	(5.5)6.0–6.6–7.3(8.2)	0.98–1.00–1.02
	SLO 2536	(5.5)6.3–6.8–7.3(8.0)	(5.5)6.2–6.8–7.3(8.0)	0.99–1.00–1.01
	SLO 2540*	(5.0)5.8–6.5–7.1(8.2)	(5.0)5.8–6.4–7.1(8.0)	0.99–1.01–1.02
	SLO 2546	(5.0)5.8–6.7–7.5(8.5)	(5.0)5.8–6.7–7.6(9.0)	0.98–1.00–1.02
	SLO 2544*	(5.5)5.9–6.6–7.2(9.0)	(5.5)5.9–6.5–7.2(9.0)	0.99–1.00–1.01
	SLO 2542	(5.0)6.0–6.9–7.8(9.0)	(5.0)6.0–6.9–7.8(9.0)	0.99–1.00–1.01
	SLO 2530*	(6.0)6.4–6.9–7.5(8.0)	(6.0)6.4–6.9–7.4(8.0)	0.99–1.00–1.02
	SLO 2526	(5.0)5.7–6.6–7.5(9.0)	(5.0)5.7–6.6–7.5(9.0)	0.99–1.00–1.02
	SLO 2527	(5.0)6.0–6.7–7.3(8.0)	(5.0)6.0–6.7–7.3(8.0)	0.98–1.00–1.02
	SLO 2529	(5.0)5.7–6.4–7.1(8.0)	(5.2)5.7–6.3–7.0(8.0)	0.99–1.00–1.02
	SLO 2518	(5.0)5.5–6.4–7.4(9.0)	(5.0)5.5–6.4–7.3(9.0)	0.99–1.01–1.02
	SLO 2519	(5.8)6.2–6.7–7.2(8.0)	(5.8)6.1–6.6–7.2(8.0)	0.99–1.00–1.02
	SLO 2520	(5.5)6.1–6.7–7.3(7.8)	(5.5)6.0–6.6–7.2(7.8)	0.99–1.00–1.02
	SLO 2541*	(5.2)6.0–6.4–6.9(7.2)	(5.2)6.0–6.4–6.8(7.2)	0.99–1.01–1.02
	SLO 1845*	(5.2)5.8–6.4–6.9(7.2)	(5.2)5.8–6.3–6.9(7.2)	0.98–1.01–1.03
	SLO 2394*	(5.5)5.9–6.7–7.6(9.2)	(5.5)5.9–6.7–7.5(9.0)	0.99–0.99–1.02
	SLO 2549	(5.5)6.0–6.8–7.7(9.0)	(5.2)6.0–6.8–7.6(9.0)	0.99–1.01–1.02
Crepidotus pini	SLO 2550*	(4.8)5.1–5.6–6.1(6.5)	(4.8)5.1–5.5–6.0(6.5)	0.99–1.01–1.04
	SLO 2601*	(5.2)5.4–5.8–6.1(6.8)	(5.2)5.3–5.7–6.0(6.5)	0.98–1.02–1.06
	SLO 2602	(5.0)5.3–5.8–6.1(7.0)	(5.0)5.3–5.7–6.1(6.8)	0.99–1.01–1.03
	SLO 2579*	(5.0)5.5–6.0–6.5(7.0)	(5.0)5.4–5.9–6.5(7.0)	0.99–1.01–1.02
	SLO 2622	(4.8)5.3–5.8–6.3(6.8)	(4.8)5.3–5.7–6.1(6.5)	0.98–1.02–1.05
	SLO 2623	(4.8)5.1–5.5–5.9(6.2)	(4.8)5.0–5.4–5.8(6.0)	0.99–1.01–1.04
