# Supplementary material for: Phylogeny of Crepidotus applanatus Look-Alikes Reveals a Convergent Morphology Evolution and a New Species C. pini
Source: J Fungi (Basel). 2022 May 7;8(5):489. doi: 10.3390/jof8050489 (PMC9144983; doi:10.3390/jof8050489)
Supplement: Supplementary file 1 [file jof-08-00489-s001.zip › Table S4.pdf]

Table S4: Estimated ranges of cheilocystidia dimensions of the studied *Crepidotus* collections based on 30 measurements represented as average (underlined) ± standard deviation. Morphological groups of cheilocystidia: cla – clavate, cyl – cylindrical, utr – utriform, ven – ventricose, for – forked, lob – lobate, pyr – pyriform, glo – globose (undifferentiated in all whether broadly or narrowly). Sequenced specimens marked with asterisk.

Taxon	Collection number	Length (µm)	Width (µm)	Morphological groups							
				cla	cyl	utr	ven	for	lob	pyr	glo
Crepidotus applanatus	SLO 1492*	24.9–31.0–37.1	7.2–9.9–12.6	20		4		5	1		
	SLO 2551*	30.9–35.0–39.1	9.2–10.5–11.7	30							
	SLO 2517	28.0–33.3–38.5	7.8–9.2–10.5	30							
	SLO 1821*	29.0–34.4–39.7	10.5–12.4–14.4	30							
	SLO 2524	31.3–38.2–45.1	8.8–11.2–13.5	28				2			
	SLO 2523	29.7–38.8–47.9	9.9–11.5–13.2	30							
	SLO 2534*	30.7–38.1–45.6	8.4–9.7–11.1	24		2		2	2		
	SLO 2535	36.3–43.0–49.6	10.2–12.1–14.0	29		1					
	SLO 2537*	32.7–37.9–43.1	8.8–10.5–12.2	22				6	2		
	SLO 2538	26.7–31.6–36.4	7.2–8.4–9.5	24		5		1			
	SLO 2539*	27.5–33.2–38.9	7.4–8.9–10.5	17			9		4		
	SLO 2533*	28.9–35.1–41.2	8.1–9.4–10.7	14		3	5	2	6		
	SLO 2545	30.5–35.7–40.9	8.2–9.7–11.2	25		2			3		
	SLO 2525	34.3–38.3–42.2	8.1–9.7–11.3	30							
	SLO 2528*	29.8–34.0–38.3	7.6–8.9–10.1	30							
	SLO 2531	30.9–37.9–44.9	7.5–8.6–9.7	21		5	4				
	SLO 2532	31.5–37.0–42.6	9.0–10.2–11.4	30							
	SLO 2547*	35.0–39.5–44.0	8.7–10.1–11.5	29	1						
	SLO 1926	27.2–32.9–38.6	5.5–9.9–14.2	28		2					
	SLO 2581*	25.6–30.6–35.6	8.6–10.0–11.4	23		3	1	2	1		
Crepidotus malachius	SLO 2552*	30.3–35.8–41.4	9.7–11.0–12.2	27			3				
	SLO 1497*	27.5–33.5–39.6	9.2–10.6–12.0	26			3	1			
	SLO 1826*	23.0–29.1–35.2	8.2–9.2–10.2	29					1		
	SLO 2548*	25.9–31.1–36.3	9.3–12.6–15.9	7			11			5	7
	SLO 479*	31.5–39.6–47.8	8.9–10.2–11.6	24			3	3			
	SLO 2091*	28.5–32.3–36.1	8.8–10.0–11.1	26		2	2				
	SLO 2536	27.2–32.2–37.2	9.0–10.5–12.0	30							
	SLO 2540*	21.5–24.5–27.5	7.7–8.9–10.1	22	1	5	2				
	SLO 2546	31.5–36.4–41.4	8.7–10.1–11.5	30							
	SLO 2544*	26.5–32.7–38.9	8.6–9.7–10.8	30							
	SLO 2542	24.9–30.8–36.7	8.6–9.8–11.0	28			2				
	SLO 2530*	24.5–28.3–32.1	8.4–10.3–12.1	30							
	SLO 2526	29.3–33.9–38.6	8.1–9.3–10.5	30							
	SLO 2527	29.3–32.5–35.7	8.6–9.5–10.4	29		1					
	SLO 2529	32.1–36.9–41.6	8.9–9.7–10.5	30							
	SLO 2518	26.4–31.5–36.5	7.6–8.7–9.7	29		1					
	SLO 2519	29.4–38.2–47.0	9.6–11.4–13.1	29		1					
	SLO 2520	27.6–33.8–40.1	8.5–9.5–10.5	29			1				
	SLO 2541*	25.3–28.9–32.5	8.1–9.0–9.8	25	1	2	2				
	SLO 1845*	28.6–32.9–37.3	8.0–9.2–10.4	25	1	2	2				
	SLO 2394*	25.0–29.0–33.0	8.6–10.2–11.7	28		1	1				
	SLO 2549	27.6–31.7–35.8	8.8–9.8–10.8	27			3				
Crepidotus pini	SLO 2550*	36.0–43.0–52.0	7.8–9.1–10.4	26		4					
	SLO 2601*	32.5–40.5–48.5	8.8–9.8–10.9	24		4		2			
	SLO 2602	38.5–45.9–53.2	8.5–10.2–11.9	20		4		1	5		
	SLO 2579*	38.8–45.7–52.6	9.4–10.6–11.7	21	1	2		2	4		
	SLO 2622	33.5–40.5–47.5	9.3–10.3–11.3	26		4					
	SLO 2623	31.2–39.0–46.7	9.5–10.6–11.6	28		2					
